# Preventable Deaths Attributable to Second-Hand Smoke in Southeast Asia—Analysis of the Global Burden of Disease Study 2019

**DOI:** 10.3389/ijph.2024.1606446

**Published:** 2024-07-04

**Authors:** Janni Leung, Carmen Lim, Tianze Sun, Giang Vu, Caitlin McClure-Thomas, Yangping Bao, Lucy Tran, Thomas Santo, Fitri Fausiah, Ghea Farassania, Gary Chung Kai Chan, Susy K. Sebayang

**Affiliations:** ^1^ National Centre for Youth Substance Use Research, School of Psychology, The University of Queensland, Brisbane, QLD, Australia; ^2^ National Institute on Drug Dependence and School of Public Health, Peking University, Beijing, China; ^3^ National Drug and Alcohol Research Centre, University of New South Wales, Kensington, NSW, Australia; ^4^ Faculty of Psychology, Department of Psychology, University of Indonesia, Depok, West Java, Indonesia; ^5^ Faculty of Health, Medicine and Life Sciences, Universitas Airlangga, Banyuwangi Campus, East Java, Indonesia

**Keywords:** mortality, passive smoking, secondhand smoke, deaths, Southeast Asia, global health, primary health care

## Abstract

**Objectives:**

In addition to harms caused to individuals who smoke, second-hand smoke (SHS or passive smoke) is an important public health issue. We aim to estimate the extent of preventable deaths due to tobacco and SHS exposure in Southeast Asia.

**Methods:**

Data were from the Global Burden of Disease Study 2019. We analysed data from Southeast Asia, including Cambodia, Indonesia, Laos, Malaysia, Maldives, Mauritius, Myanmar, Philippines, Seychelles, Sri Lanka, Thailand, Timor-Leste, and Vietnam.

**Results:**

In 2019, there were 728,500 deaths attributable to tobacco in Southeast Asia, with 128,200 deaths attributed to SHS exposure. The leading causes of preventable deaths were ischemic heart disease, stroke, diabetes mellitus, lower respiratory infections, chronic obstructive pulmonary disease, tracheal, bronchus, and lung cancer. Among deaths attributable to tobacco, females had higher proportions of deaths attributable to SHS exposure than males in Southeast Asia.

**Conclusion:**

The burden of preventable deaths in a year due to SHS exposure in Southeast Asia is substantial. The implementation and enforcement of smoke-free policies should be prioritized to reduce the disease burden attributed to passive smoking in Southeast Asia.

## Introduction

Tobacco use ranks as a top global risk factor for disease burden, attributable not only to the direct impact of smoking but also to exposure to second-hand smoke (SHS) [1–3]. Existing reviews suggest that SHS is associated with an increased risk of severe health conditions including respiratory diseases [4], cardiovascular diseases [5], and lung cancer [6]. In 2019, the World Health Organization (WHO) Global Burden of Disease Study estimated that SHS accounted for 37.0 million disability-adjusted life years (DALYs) and led to 1.3 million deaths globally [7], emphasizing the critical need to address SHS exposure as a key public health priority.

Southeast Asia region has one of the highest rates of tobacco consumption [8] and an associated health burden globally. It was estimated that approximately 1 in 3 people aged 15 years or older living in this region currently use tobacco [8], contributing to over 3.9 million DALYs and 128,000 deaths [7]. Men were disproportionately more likely to smoke in this region (43.7%) than women (9.4%) [8], which could indirectly contribute to significant second smoke exposure among non-smokers, including vulnerable populations such as children and women. Addressing harms related to tobacco use in Southeast Asia is particularly complicated because it is also a major area for tobacco production [9], further complicating prevention efforts to reduce tobacco use. Therefore, rules and policies to create smoke-free environments are required to reduce exposure to SHS [10, 11]. All Southeast Asian countries have implemented smoke-free policies; however, the policy coverage in some countries may not be comprehensive. While smoking is universally banned in healthcare, education, and government facilities across most countries, places such as private offices, public transport, restaurants, and pubs still allow smoking to various extent from designated smoking areas to no restriction [12]. Challenges also exists in terms of compliance. For example, although smoking is prohibited in most public places and areas frequented by children, compliance with smoke-free laws remained low [13].

Only two recent studies [1, 3] have examined the disease burden of SHS harm on the global level. Su et al [1] focused on analyzing the global rate of change in age standardized prevalence and quantifying mortality associated with SHS in China, India and worldwide. Zhai et al [3] estimated the pooled global DALY, YLDs, and YLLs attributable to SHS. Neither study has focused on preventable deaths specifically caused by SHS nor did they report country-specific estimates for Southeast Asia, an important indicator for measuring progress towards the WHO Framework Convention on Tobacco Control and to identify priority areas.

This study aims to 1) estimate the number and proportion of deaths attributable to SHS in females and males in Southeast Asian countries and 2) investigate the rates and number of preventative deaths attributable to SHS caused by specific disease using the Global Burden of Disease study.

## Methods

### Design

Data were from the 2019 Global Burden of Disease, Injuries, and Risk Factors (GBD) Study. The GBD study is an ongoing effort to quantify the impact of 369 diseases and injuries and 87 risk factors covering 21 regions and 204 countries and territories around the world. The study provides a comprehensive assessment of incidence, prevalence, mortality, disability, and burden of diseases across countries, age-groups and gender. A four-tier hierarchical structure was used to categorise causes of death and disability where level 1 causes is the broadest category of all and it encompass non-communicable diseases, injuries and a category that includes communicable, maternal, neonatal, and nutritional diseases and impairments. Level 2 causes encompass 22 diseases and injuries, such as neurological disorders, substance use disorders, and unintentional injuries, while level 3 and 4 causes are more specific [14].

### Data Sources

Data on the disease burden attributable to tobacco and SHS, including cases and rates of deaths across all ages and their 95% uncertainty intervals were extracted from the Global Health Data Exchange (GHDx) database [15]. The data source for the GBD study came from systematic reviews, external searches on government and international websites, published reports, existing surveys, and contributions of datasets by GBD collaborators.

### Exposure to Second-Hand Smoke

#### Case Definition

The GBD study define SHS exposure as exposure to second-hand tobacco smoke across various settings such as home, work, or public places. Household composition was used as a proxy of non-occupational SHS exposure where individuals of the general population across all ages living with a person that smokes daily is assumed to be exposed to tobacco smoke. Additionally, surveys were also used to estimate the proportion of non-smokers exposed to SHS at work. Non-smokers would include individuals who are not smoking daily.

#### Input Data

The percentage of individuals who did not smoke living with one who smoked was derived using household composition data, which included the age and sex of all individuals residing in the same dwelling. GBD analysed data for females and males, so these terms are used to refer to the sex categories in the results. The data were collected from a series of reputable surveys (e.g., Demographic Health Surveys, the Multiple Indicator Cluster Surveys, and the Living Standards Measurement Surveys) that contained a module on household composition and from national and sub-national census. The proportion of individuals exposed to SHS at work (by age and sex) was derived using cross-sectional surveys that collect information on self-reported occupational SHS exposure. The list of GBD 2019 data input sources for tobacco exposure and second-hand smoke exposure as risk factors in Southeast Asia is presented in Supplementary Table S3.

In summary, 62 sources (n = 7,856 data-points) across 13 countries in Southeast Asia analysed in the GBD study (Laos, Myanmar, Philippines, Thailand, Timor-Leste, Vietnam, Cambodia, Indonesia, Maldives, Mauritius, Sri Lanka, Seychelles, and Malaysia) were utilised for calculating risk factor of second-hand smoking on the diseases [16].

The specific disease outcomes analysed include ischemic heart disease, stroke, chronic obstructive pulmonary disease, diabetes, cancer, and respiratory infections [14]. The detailed data sources used to estimate and compute the burden of second-hand smoking on disease across Southeast Asian countries can be found using the GBD 2019 Data Input Sources Tool.

#### Modelling Strategy

Preventable deaths attributable to second-hand smoke in Southeast Asia in 2019 were estimated. We focused on the year of 2019 because it is the most recent estimation available, so it would the closest insight for evaluation of the current status. The estimation of attributable burden adhered to the framework for comparative risk assessment (CRA) [17]. This framework constructs a hierarchical causal web of risks or causes that contributes to health outcomes. Like the causes of death and disability, these risks were organised into 4 hierarchical levels where level 1 includes broad categories of risk factors (e.g., behavioural risks, occupational risks) and level 4 provides the most detailed risk information (e.g., stunting, underweight). SHS is a level 3 risk factor. A key element of the CRA is the identification of the risk-outcome pairs for the considered risk factor(s). A risk factor can be conceived as any exposure that increases the risk of developing a certain health outcome, which involves evidence establishment of causality. This is based on the strength of evidence in existing literature. The GBD study uses the World Cancer Research Fund criteria for “convincing or probable evidence” to include risk-outcome pairs, adapted from the Bradford-Hill criteria. For example, for the risk-outcome-pair of ambient particulate matter and lung cancer, evidence for a causal link have been shown consistently in many studies, including epidemiological and toxicological assessments.

Attributable burden was estimated using steps laid out in the CRA framework [18]. The CRA is to compare a current harmful risk factor exposure level in the population against an alternative (or “counterfactual”) exposure situation where the selected risk factor is reduced to the so-called Theoretical Minimum Risk Exposure Level (TMREL). For example, the TMREL could correspond to zero second-hand smoke exposure. The six steps of the CRA are: I) inclusion of risk-outcome pairs in the analysis, ii) estimating relative risk (RR) as a function of exposure, iii) estimating exposure levels and distributions, iv) determining the counterfactual level of exposure (known as theoretical minimum risk exposure level (TMRELs), v) calculating population attributable fractions (PAFs) and attributable burden, and vi) estimating the mediation of different risk factors through other risk factors.

Attributable burden of SHS is defined as the amount of reduction in the specific disease burden that could be prevented if second hand smoking was eliminated. Briefly, the probability of non-smokers living with someone who smoked and the probability of being a non-smoker themselves were estimated using set theory. As there was no difference in the household composition between those who smoked and those who did not, the probability that each household has a person who smoked daily was calculated using the GBD 2019 primary daily smoking prevalence model. Then the overall probability that at least one of the household members who smoked daily was derived based on the probability of the union of sets on each individual household member. Occupational exposure was incorporated by modelling the current prevalence of SHS exposure at work based on age, gender, location, and year. The probability of exposure was multiplied by the probability of the individual being a non-smoker themselves. Finally individual-level probabilities were aggregated to derive the average probabilities of exposure by location, year, age, and sex. These probabilities were modelled using the spatiotemporal Gaussian process regression model (ST-GPR). The standard GBD population attributable fraction equation was used to estimate attributable burden of SHS based on exposure and relative risks. Specific details of the methodology are described elsewhere [18].

Cases and rates of deaths, by sex along with the 95% uncertainty intervals (UIs) attributable to tobacco and SHS in Southeast Asian countries were described and tabulated. Next, the number of preventable deaths attributable to SHS by specific disease cause was summarised using a treemap. The proportion of deaths attributable to SHS among deaths attributable to tobacco is also calculated.

## Results

### Deaths Attributable to Tobacco and Second-Hand Smoke

In Southeast Asia in 2019, the overall cases of deaths that were attributable to using tobacco was 728,500 [95%UI = 655,400–803,600] with and rate of 108.1 [97.3–119.3] per 100 k (see Table 1).

**TABLE 1:** All ages rates and cases of deaths attributable to tobacco and second-hand smoke in women and men in Southeast Asian countries in 2019.

			Deaths attributable to tobacco overall		Deaths attributable to second-hand smoke specifically	
			All age rates per 100 k	Cases (rounded to 100)	All age rates per 100 k	Cases (rounded to 100)
**Both sex (Total)**						
	**Southeast Asia**		**108.1 [97.3-119.3]**	**728,500 [655,400-803,600]**	**19.0 [14.7-23.4]**	**128,200 [98,800-157,500]**
		Cambodia	116.7 [94.9–133.1]	19,400 [15,800–22,100]	20.3 [15.0–25.8]	3,400 [2,500–4,300]
		Indonesia	111.9 [92.3–130.6]	290,400 [239,400–338,900]	20.3 [15.1–25.5]	52,600 [39,300–66,300]
		Laos	97.9 [79.0–117.1]	7,000 [5,700–8,400]	19.3 [14.0–25.2]	1,400 [1,000–1,800]
		Malaysia	94.1 [76.0–116.1]	29,500 [23,800–36,300]	19.1 [13.2–25.5]	6,000 [4,100–8,000]
		Maldives	48.4 [40.9–56.4]	200 [200–300]	9.2 [6.9–11.9]	<100
		Mauritius	108.8 [87.4–134.0]	1,400 [1,100–1,700]	30.8 [20.5–42.4]	400 [300–500]
		Myanmar	125.5 [109.3–146.4]	68,600 [59,800–80,100]	19.9 [14.4–26.0]	10,900 [7,900–14,200]
		Philippines	100.0 [81.8–121.6]	112,100 [91,700–136,400]	19.3 [14.2–25.0]	21,700 [15,900–28,000]
		Seychelles	118.5 [103.8–134.2]	100 [100–100]	18.7 [13.7–23.9]	<100
		Sri Lanka	64.9 [49.2–84.7]	14,200 [10,800–18,500]	13.9 [9.1–19.7]	3,000 [2,000–4,300]
		Thailand	101.2 [76.2–130.6]	71,000 [53,400–91,600]	13.5 [9.1–18.9]	9,400 [6,400–13,200]
		Timor-Leste	82.2 [64.7–98.3]	1,100 [900–1,300]	15.2 [10.6–20.2]	200 [100–300]
		Viet Nam	116.8 [98.0–135.4]	112,600 [94,500–130,500]	19.8 [14.9–25.2]	19,100 [14,400–24,300]
**Male**						
	**Southeast Asia**		**172.1 [153.0-191.2]**	**579,300 [515,200-643,700]**	15.3 [11.4–19.4]	**51,600 [38,500-65,300]**
		Cambodia	179.4 [145.8–203.4]	14,600 [11,900–16,600]	13.7 [9.7–18.0]	1,100 [800–1,500]
		Indonesia	178.8 [144.9–217.2]	234,000 [189,700–284,300]	13.5 [9.3–17.9]	17,600 [12,200–23,500]
		Laos	152.4 [123.1–181.6]	5,500 [4,400–6,500]	15.3 [10.7–20.6]	600 [400–700]
		Malaysia	151.1 [121.1–186.7]	24,500 [19,600–30,200]	17.1 [11.6–23.3]	2,800 [1,900–3,800]
		Maldives	65.1 [55.5–75.6]	200 [200–200]	7.7 [5.8–10.0]	<100
		Mauritius	169.3 [136.9–206.6]	1,100 [900–1,300]	26.1 [17.5–36.5]	200 [100–200]
		Myanmar	182.4 [158.7–213.9]	48,000 [41,700–56,200]	19.0 [13.4–25.7]	5,000 [3,500–6,800]
		Philippines	149.5 [116.4–192.6]	85,000 [66,200–109,500]	19.3 [13.5–26.0]	11,000 [7,700–14,800]
		Seychelles	187.6 [162.3–215.6]	100 [100–100]	17.3 [12.4–22.2]	<100
		Sri Lanka	106.7 [80.8–138.8]	11,300 [8,500–14,700]	12.0 [7.8–17.3]	1,300 [800–1,800]
		Thailand	170.7 [127.7–220.7]	58,400 [43,700–75,500]	11.9 [7.7–17.0]	4,100 [2,600–5,800]
		Timor-Leste	133.7 [102.8–159.9]	900 [700–1,100]	13.2 [8.9–17.9]	100 [100–100]
		Viet Nam	199.1 [166.6–231.1]	95,000 [79,500–110,300]	16.5 [12.1–21.4]	7,900 [5,800–10,200]
**Female**						
	**Southeast Asia**		**44.3 [38.1-50.9]**	**149,200 [128,400-171,600]**	**22.7 [17.2-28.1]**	**76,600 [58,200-94,900]**
		Cambodia	56.2 [45.6–66.0]	4,700 [3,900–5,600]	26.7 [19.9–33.9]	2,300 [1,700–2,900]
		Indonesia	43.9 [34.2–54.4]	56,400 [43,900–69,900]	27.2 [20.0–35.5]	34,900 [25,700–45,700]
		Laos	42.8 [34.0–53.1]	1,500 [1,200–1,900]	23.4 [17.1–30.6]	800 [600–1,100]
		Malaysia	33.0 [25.6–42.1]	5,000 [3,900–6,400]	21.2 [14.9–28.3]	3,200 [2,300–4,300]
		Maldives	23.3 [18.8–28.5]	<100	11.3 [8.5–14.6]	<100
		Mauritius	49.9 [36.2–65.0]	300 [200–400]	35.4 [23.0–48.8]	200 [100–300]
		Myanmar	72.8 [61.8–85.1]	20,700 [17,500–24,100]	20.7 [15.2–27.0]	5,900 [4,300–7,700]
		Philippines	49.0 [38.4–62.0]	27,100 [21,200–34,300]	19.4 [13.7–26.0]	10,700 [7,600–14,400]
		Seychelles	41.8 [33.7–50.5]	<100	20.3 [14.9–26.1]	<100
		Sri Lanka	25.9 [18.7–34.4]	2,900 [2,100–3,900]	15.6 [10.2–22.3]	1,800 [1,100–2,500]
		Thailand	35.0 [26.3–45.8]	12,600 [9,400–16,400]	15.0 [10.1–21.0]	5,400 [3,600–7,500]
		Timor-Leste	29.4 [22.3–37.0]	200 [100–200]	17.2 [12.1–22.6]	100 [100–100]
		Viet Nam	36.1 [29.0–44.3]	17,500 [14,100–21,500]	23.0 [17.3–29.4]	11,200 [8,400–14,300]

*Note*. Cases are rounded to the nearest 100; 95% uncertainty intervals are presented in parentheses. The bold values indicate overall estimates for the whole South East Asian region.

The overall deaths attributable to SHS in Southeast Asia in 2019 was 128,200 [98,800–157,500] total cases with an all ages rate of 19.0 [14.7–23.4] per 100 k (see Table 1). The country in the analysis that had the lowest rates per 100 k for SHS deaths was Maldives (9.2 [6.9–11.9], total cases= <100), the highest was Mauritius (30.8 [20.5–42.4] per 100 k, cases = 400 [300–500]). The countries that had the lowest cases of deaths attributable to SHS was Seychelles and Maldives as they both had fewer than 100 cases. Indonesia had the highest cases of deaths attributable to SHS, 52,600 [39,300–66,300] cases and a mortality rate per 100 k of 20.3 [15.1–25.5].

Of that, the overall deaths attributable to SHS in females were 76,600 [58,200–94,900] cases with a rate of 22.7 [17.2–28.1], compared to males who had overall 51,600 [38,500–65,300] death cases with a 15.3 [11.4–19.4] death rate that was attributable to SHS.

For females, the country with the lowest death rate for attributable SHS deaths was Maldives with a rate of 11.3 [8.5–14.6] (total cases= <100). The country with the highest rate for females was Mauritius (35.4 [23.0–48.8], cases = 200 [100–300]). The country with the lowest cases of SHS deaths in females was Seychelles and Maldives as they both had less than 100 cases. The country with the highest cases was Indonesia (34,900 [25,700–45,700], 27.2 [20.0–35.5] rate per 100 k).

Males were comparably lower than female in cases and rates of deaths attributable to SHS. The country with the lowest rate of deaths for SHS in males was Maldives, 7.7 [5.8–10.0] rate (cases= <100) and the highest was Mauritius with a rate of 26.1 [17.5–36.5] (cases = 200 [100–200]). The country with the lowest cases of SHS deaths for males was Seychelles and Maldives as they both had less than 100 cases. The country with the highest deaths cases for males was 17,600 [12,200–23,500] (13.5 [9.3–17.9] rate per 100 k) in Indonesia.

### Causes of Deaths Attributable to Second-Hand Smoke

**FIGURE 1:**
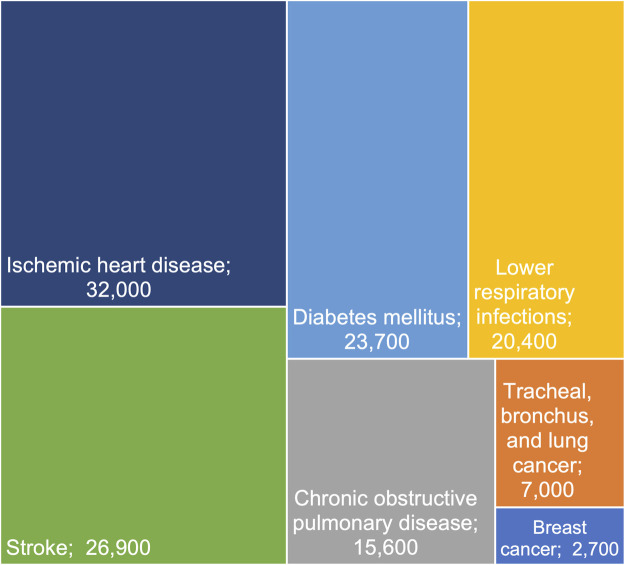
Preventable deaths attributable to second-hand smoke by specific disease causes in Southeast Asia in 2019. *Note.* Cases are rounded to the nearest 100. Uncertainty intervals and numbers in males and females are available in Supplementary Table S1.

Figure 1 presents the estimated number of preventable deaths (in hundreds) attributable to SHS by specific disease causes in Southeast Asia in 2019 (see Supplementary Material S1 for data table). The leading cause of death attributable to SHS was ischemic heart disease (32,000 [25,500–38,800]), which caused 13,700 [10,500–17,100] male deaths and 18,300 [14,100–22,500] female deaths. This was followed by stroke (total = 26,900 [19,500–35,100], male = 9,600 [6,700–12,800], female = 17,300 [12,500–22,800]), and diabetes mellitus (total = 23,700 [9,600–36,400], male = 9,100 [4,900–13,600], female = 16,400 [6,700–24,900]). The fourth leading cause of deaths from SHS was lower respiratory infections (total = 20,400 [11,200–30,200], male = 8,300 [4,000–13,600], female = 11,300 [6,200–16,800]). Next were chronic obstructive pulmonary disease (total = 15,600 [7,600–24,800], male = 7,300 [2,700–11,600], female = 7,300 [3,400–11,400]), and tracheal, bronchus, and lung cancer (total = 7,000 [3,800–11,000], male = 3,600 [2,000–5,700], female = 3,400 [1,800–5,600]). An estimated number of 2,700 [600–4,700] deaths caused by breast cancer in females were attributable to SHS in Southeast Asia.

### Proportion of Deaths Attributable to Second-Hand Smoke Among Deaths From Tobacco by Sex

**FIGURE 2:**
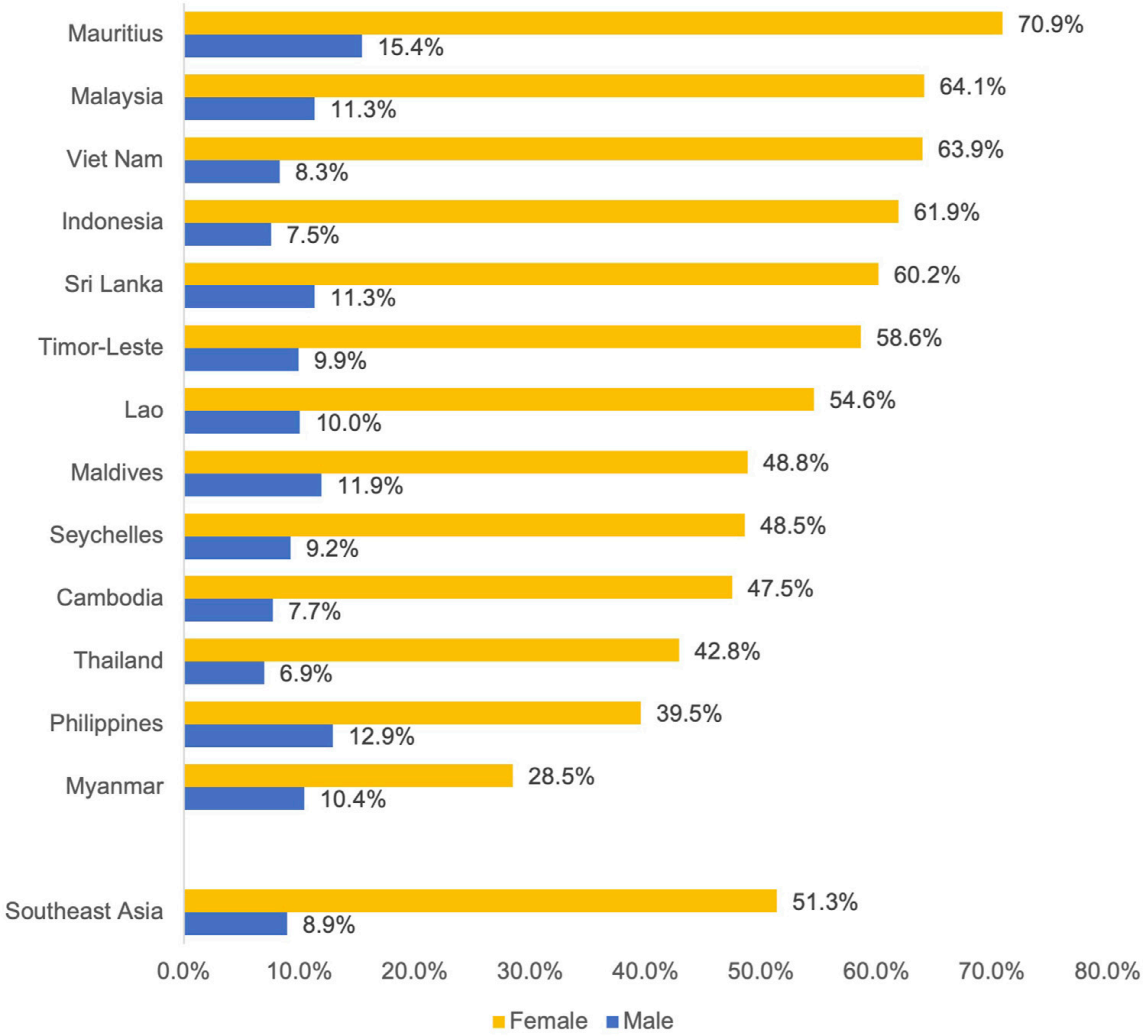
Proportion of deaths attributable to second-hand smoke among deaths attributable to tobacco in 2019. *Note.* Uncertainty intervals are available in Supplementary Table S2.

The proportion of deaths attributable to SHS among deaths attributable to tobacco in 2019 was evaluated (see Figure 2; Supplementary Material S2). For males in Southeast Asia the proportion of deaths attributable to SHS was 8.9% (7.5%–10.1%). For females in Southeast Asia the proportion was 51.3% (45.3%–55.3%). The country with the lowest proportion of deaths attributable to SHS for males was Thailand, 6.9% (6.0%–7.7%) and Myanmar, 28.5% (24.6%–31.8%) had the lowest proportion for females. The country with the highest proportion of deaths attributable to SHS for males was Mauritius, 15.4% (12.8%–17.7%) and the highest for females was also Mauritius for a proportion of deaths equalling 70.9% (63.6%–75.1%).

## Discussion

Our study found that tobacco use was responsible for 728,500 deaths in Southeast Asia, with 128,200 deaths attributed to SHS. Death rates and cases varied by country, with Mauritius having the highest death rate (30.8) and Maldives having the lowest (9.2). Females had higher rates than males in comparison. The countries with the lowest number of SHS deaths were Seychelles and Maldives, with fewer than 100 deaths. This trend was consistent for both males and females. Indonesia had the highest number of deaths attributable to SHS (52,600) and the highest number of SHS deaths for both males (17,600) and females (34,900). Overall, females had a higher number of deaths attributable to SHS.

Our study has shown a significant burden of preventable deaths due to SHS exposure in Southeast Asia, with the leading causes of death being ischemic heart disease, stroke, diabetes mellitus, lower respiratory infections, chronic obstructive pulmonary disease, tracheal, bronchus, and lung cancer. We also found a considerable number of breast cancer deaths in females attributable to SHS exposure in the region.

Our study found that more females than males died from SHS attributable to tobacco, both overall and in each country in Southeast Asia. Thailand had the lowest proportion of SHS deaths among males, while Myanmar had the lowest proportion of deaths among females. Mauritius had the highest proportion of deaths attributable to SHS among tobacco deaths in both males and females. In Southeast Asia, compared to other countries, there is a greater gap in smoking prevalence by gender, with men being more likely to smoke, whereas women are more likely to be exposed to SHS, and therefore, develop tobacco-related harm. SHS-attributable specific burden is higher in Southeast Asian women than the burden directly attributed to smoking tobacco [19].

It is important to consider both death rates and absolute number of cases when assessing the impact of SHS exposure. The use of rate data provides a more accurate representation of the relative burden of SHS exposure in countries with a low number of cases due to small population sizes. Therefore, high rates of the relative burden of SHS exposure were detected in Cambodia and Seychelles. Conversely, countries with both high rates and cases, such as Vietnam or Indonesia, can use both metrics to guide their efforts in addressing SHS.

A lack of knowledge and awareness about the complications of SHS acts as a barrier to avoiding exposure to SHS [20]. The existing literature suggests variability in the level of knowledge of SHS-related health issues across countries, as well as among different sub-population groups in Southeast Asia. For example, approximately 89% of school children [21] and 90% of adults [22] in Vietnam are aware of the harmful effects of cigarette smoke. Another study of high school students found that while most students (99%) knew about the harms of tobacco, very few (25%) knew about tobacco product laws [23]. Several studies also found that higher educated groups may possess a higher level of knowledge of SHS compared to the general population [24, 25]. A limited number of studies on SHS avoidance showed a significant correlation between knowledge of SHS harm and the likelihood of avoiding SHS exposure [24, 26, 27]. Improved knowledge can empower individuals to make informed decisions about their health and minimize the risks associated with SHS exposure. Thus, enhancing health literacy on SHS- and tobacco-related effects, possibly through education campaigns, should be considered.

Our results showed that a substantial number of females in Southeast Asia died from SHS exposure, regardless of whether they smoked. This highlights the need for more comprehensive smoke-free laws throughout the region (i.e., smoking to be banned completely from all public areas, restaurants, and workplaces). Exposure to SHS at home should also be addressed, since people who smoke and those affected by their smoking generally spend a large proportion of their time in this environment. However, avoiding SHS exposure at home may not be effective or feasible within a generally small home space. Thus, it is important for women to be empowered to take actions that can minimize and eliminate smoking at home. A recent review found that SHS campaigns can be effective in reducing smoking behaviour in homes and around children [28], underscoring the potential impact of targeted public health interventions. As Southeast Asian countries are significantly diverse in their cultural context and the role of women in their societies [29], this calls for context-specific public health interventions aimed at not only educating women on the harms of SHS, but also empowering them and providing them with appropriate methods to effectively avoid SHS exposure.

Studies have begun to empower women to contribute to the prevention of SHS exposure. For example, a study examining the impact of a multicomponent intervention on household SHS exposure among Sri Lankan women found that knowledge of the health risks of exposure to SHS, attitudes of women towards exposure to SHS, right to smoke free living, and women empowerment against smoking had significantly improved among women in the intervention group compared to the control group after the intervention [30]. Such measures would ensure non-smoking women’s rights to enjoy the highest attainable standard of physical and mental health.

Our study also found that athough small compared to SHS-attributable mortality, deaths in women attributed to active smoking are still substantial. A recent study estimated the prevalence of current smoking in women globally to be 17%, while that of ever smoking in Asian women was 14% [31], although a decreasing smoking trend has been reported elsewhere [32]. Smoking cessations are thus needed for women as much as they are for men. As factors that can influence success in quitting smoking vary by gender, it is important that public health interventions for smoking cessation are developed and delivered with a sex-specific mindset.

The United Nation’s Sustainable Development Goals (SDG) call for all countries to ensure healthy lives and promote well-being for all [33], in which a key target is to strengthen the implementation of the World Health Organization Framework Convention on Tobacco Control (WHO FCTC) [34]. Protection from exposure to tobacco smoke is one of the main objectives of the WHO FCTC [34]. Smoke-free policies and rules can address these goals because they are effective in protecting the public from passive smoke [35]. This study showed that a high number of deaths in Southeast Asia are attributable to exposure to tobacco smoking, which provides evidence that the implementation and enforcement of smoke-free policies should be prioritised to prevent deaths due to passive smoking in public, workplaces, or at home.

### Limitations

The limitations of this study aligns with those outlined in the GBD 2019 study. The main limitation of the GBD study is the availability and accessibility of primary data, which can vary significantly across regions. This poses a major challenge in accurately estimating the burden of disease in any given population. In instances where primary data was not accessible, the results relied on out-of-sample predictive validity of the statistical models. Although this resulted in marginal improvements in the precision of the estimates, there is a pressing need for more primary data. Data may not have been collected using the optimal case definition or the measurement method. However, this was improved through the identification of desired and alternative measurement methods for each outcome along with the process of mapping biases from alternative to reference methods. The GBD study also utilise ‘standard location’ in their fixed effects model to increase the stability of models between cycles, although collinearity between covariates could still contribute to instability in fixed effects between cycles. The cause of death models, an ensemble model was developed to help address the collinearity issue. The uncertainty intervals (UI) especially in locations with limited or no data could be less accurate although the statistical model is designed to capture uncertainty from stochastic variation in input data.

### Conclusion

In Southeast Asia, 728.5 thousand deaths were attributable to tobacco, of which 128.2 thousand deaths were attributable to SHS specifically. The top three causes of these preventable deaths were from heart disease, stroke, and diabetes. Over half of the deaths attributed to tobacco in women in the region could have been avoided if they were not exposed to SHS. Public health interventions to create smoke-free environments should be prioritised to reduce the disease burden attributed to SHS in Southeast Asia.
