# Supplementary material for: Preventable Deaths Attributable to Second-Hand Smoke in Southeast Asia—Analysis of the Global Burden of Disease Study 2019
Source: Int J Public Health. 2024 Jul 4;69:1606446. doi: 10.3389/ijph.2024.1606446 (PMC11254616; doi:10.3389/ijph.2024.1606446)
Supplement: Supplementary file 1 [file DataSheet1.docx]

# **Supplementary materials**

Preventable deaths attributable to second-hand smoke in Southeast Asia – analysis of the Global Burden of Disease Study 2019

### **S1. Data table for Figure 1 - Preventable deaths attributable to second-hand smoke by specific disease causes in Southeast Asia in 2019**

|  |  | **Number of deaths attributable to second-hand smoke** | | |
| --- | --- | --- | --- | --- |
|  |  | **Cases  (in 100s)** | **95% UI** | |
| **Causes** |  |  | **lower** | **upper** |
| **Both sex (Overall)** | |  |  |  |
|  | Ischemic heart disease | 32,000 | 25,500 | 38,800 |
|  | Stroke | 26,900 | 19,500 | 35,100 |
|  | Diabetes mellitus | 23,700 | 9,600 | 36,400 |
|  | Lower respiratory infections | 20,400 | 11,200 | 30,200 |
|  | Chronic obstructive pulmonary disease | 15,600 | 7,600 | 24,800 |
|  | Tracheal, bronchus, and lung cancer | 7,000 | 3,800 | 11,000 |
|  | Breast cancer | 2,700 | 600 | 4,700 |
|  | Otitis media | <100 | <100 | <100 |
| **Male** |  |  |  |  |
|  | Ischemic heart disease | 13,700 | 10,500 | 17,100 |
|  | Stroke | 9,600 | 6,700 | 12,800 |
|  | Lower respiratory infections | 9,100 | 4,900 | 13,600 |
|  | Chronic obstructive pulmonary disease | 8,300 | 4,000 | 13,600 |
|  | Diabetes mellitus | 7,300 | 2,700 | 11,600 |
|  | Tracheal, bronchus, and lung cancer | 3,600 | 2,000 | 5,700 |
|  | Breast cancer | <100 | <100 | <100 |
|  | Otitis media | <100 | <100 | <100 |
| **Female** |  |  |  |  |
|  | Ischemic heart disease | 18,300 | 14,100 | 22,500 |
|  | Stroke | 17,300 | 12,500 | 22,800 |
|  | Diabetes mellitus | 16,400 | 6,700 | 24,900 |
|  | Lower respiratory infections | 11,300 | 6,200 | 16,800 |
|  | Chronic obstructive pulmonary disease | 7,300 | 3,400 | 11,400 |
|  | Tracheal, bronchus, and lung cancer | 3,400 | 1,800 | 5,600 |
|  | Breast cancer | 2,700 | 600 | 4,700 |
|  | Otitis media | <100 | <100 | <100 |
| *Note.* UI, uncertainty intervals | | | | |

### **S2. Data table for Figure 2 - Proportion of deaths attributable to second-hand smoke among deaths attributable to tobacco in 2019**

|  |  |  | **Proportion attributable to  second-hand smoke  among deaths from tobacco** | | |
| --- | --- | --- | --- | --- | --- |
|  |  |  | **%** | **lower** | **upper** |
| **Male** |  |  |  |  |  |
|  | **Southeast Asia** | | 8.9% | 7.5% | 10.1% |
|  |  | Cambodia | 7.7% | 6.6% | 8.9% |
|  |  | Indonesia | 7.5% | 6.4% | 8.3% |
|  |  | Laos | 10.0% | 8.7% | 11.4% |
|  |  | Malaysia | 11.3% | 9.6% | 12.5% |
|  |  | Maldives | 11.9% | 10.4% | 13.2% |
|  |  | Mauritius | 15.4% | 12.8% | 17.7% |
|  |  | Myanmar | 10.4% | 8.5% | 12.0% |
|  |  | Philippines | 12.9% | 11.6% | 13.5% |
|  |  | Seychelles | 9.2% | 7.7% | 10.3% |
|  |  | Sri Lanka | 11.3% | 9.7% | 12.5% |
|  |  | Thailand | 6.9% | 6.0% | 7.7% |
|  |  | Timor-Leste | 9.9% | 8.7% | 11.2% |
|  |  | Viet Nam | 8.3% | 7.3% | 9.3% |
| **Female** | |  |  |  |  |
|  | **Southeast Asia** | | 51.3% | 45.3% | 55.3% |
|  |  | Cambodia | 47.5% | 43.7% | 51.3% |
|  |  | Indonesia | 61.9% | 58.4% | 65.3% |
|  |  | Laos | 54.6% | 50.4% | 57.6% |
|  |  | Malaysia | 64.1% | 58.2% | 67.2% |
|  |  | Maldives | 48.8% | 45.2% | 51.2% |
|  |  | Mauritius | 70.9% | 63.6% | 75.1% |
|  |  | Myanmar | 28.5% | 24.6% | 31.8% |
|  |  | Philippines | 39.5% | 35.8% | 42.0% |
|  |  | Seychelles | 48.5% | 44.1% | 51.7% |
|  |  | Sri Lanka | 60.2% | 54.3% | 64.8% |
|  |  | Thailand | 42.8% | 38.4% | 45.8% |
|  |  | Timor-Leste | 58.6% | 54.4% | 60.9% |
|  |  | Viet Nam | 63.9% | 59.6% | 66.5% |
| *Note.* UI, uncertainty intervals | | | | | |

### **S3. Original sources for data on second-hand smoke exposure risk factor in Southeast Asia in the GBD 2019 study**

| GBD 2019 data input sources for tobacco risk factor, Southeast Asia |
| --- |
| Macro International, Inc, Ministry of Health (Cambodia), National Institute of Statistics (Cambodia). Cambodia Demographic and Health Survey 2000. Fairfax, United States of America: ICF International |
| Macro International, Inc, National Institute of Public Health (Cambodia), National Institute of Statistics (Cambodia). Cambodia Demographic and Health Survey 2005-2006. Fairfax, United States of America: ICF International |
| Centers for Disease Control and Prevention (CDC) and World Health Organization (WHO). Cambodia Global Youth Tobacco Survey 2003. United States: Centers for Disease Control and Prevention (CDC), 2003 |
| ICF Macro, Ministry of Health (Cambodia), National Institute of Statistics (Cambodia). Cambodia Demographic and Health Survey 2010-2011. Fairfax, United States of America: ICF International |
| National Institute of Statistics (Cambodia), United Nations Development Programme (UNDP), World Bank. Cambodia Socio-Economic Survey 1999. Phnom Penh, Cambodia: National Institute of Statistics (Cambodia) |
| National Institute of Statistics (Cambodia), Statistics Sweden. Cambodia Socio-Economic Survey 2003-2005. Phnom Penh, Cambodia: National Institute of Statistics (Cambodia) |
| National Institute of Statistics (Cambodia), Statistics Sweden. Cambodia Socio-Economic Survey 2006-2007. Phnom Penh, Cambodia: National Institute of Statistics (Cambodia) |
| Ministry of Health (Cambodia), University of Health Sciences (Cambodia), World Health Organization (WHO). Cambodia STEPS Noncommunicable Disease Risk Factors Survey 2010 |
| Centers for Disease Control and Prevention (CDC), World Health Organization (WHO). Cambodia Global Youth Tobacco Survey 2010. Atlanta, United States of America: Centers for Disease Control and Prevention (CDC) |
| National Institute of Statistics (Cambodia). Cambodia Smoking Behavior Survey 2004 |
| Institute for Social Research, University of Michigan. Cambodia Elderly Survey 2004. Ann Arbor, United States of America: Institute for Social Research, University of Michigan |
| Centers for Disease Control and Prevention (CDC), Ministry of Health (Cambodia), World Health Organization (WHO). Cambodia Global School-Based Student Health Survey 2013. Atlanta, United States of America: Centers for Disease Control and Prevention (CDC) |
| ICF International, Ministry of Health (Cambodia), National Institute of Statistics (Cambodia). Cambodia Demographic and Health Survey 2014. Fairfax, United States of America: ICF International, 2017 |
| U.S. Department of Agriculture (USDA). USDA Global Tobacco Database 1960-2005. Washington D.C. , United States: U.S. Department of Agriculture (USDA) |
| Food and Agriculture Organization of the United Nations (FAO). FAOSTAT Commodity Balances - Crops Primary Equivalent. Rome, Italy: Food and Agriculture Organization of the United Nations (FAO) |
| Gallup. Cambodia World Poll 2005-2006 |
| Gallup. Cambodia World Poll 2011 |
| Gallup. Cambodia World Poll 2012 |
| Centers for Disease Control and Prevention (CDC), Ministry of Health (Cambodia), World Health Organization (WHO). Cambodia Global Youth Tobacco Survey 2016. Atlanta, United States of America: Centers for Disease Control and Prevention (CDC) |
| World Health Organization (WHO). WHO Report on the Global Tobacco Epidemic 2019. Geneva, Switzerland: World Health Organization (WHO), 2019 |
| RAND Corporation, University of Indonesia. Indonesia Family Life Survey 1993-1994. Santa Monica, United States of America: RAND Corporation |
| Macro International, Inc, RAND Corporation, University of California, Los Angeles (UCLA), University of Indonesia. Indonesia Family Life Survey 1997 |
| Center for Population and Policy Studies, Gadjah Mada University (Indonesia), RAND Corporation. Indonesia Family Life Survey 2000. Santa Monica, United States of America: RAND Corporation |
| Center for Population and Policy Studies, Gadjah Mada University (Indonesia), RAND Corporation, SurveyMETER. Indonesia Family Life Survey 2007-2008. Santa Monica, United States of America: RAND Corporation |
| Centers for Disease Control and Prevention (CDC) and World Health Organization (WHO). Indonesia Global Youth Tobacco Survey 2006. Atlanta, United States: Centers for Disease Control and Prevention (CDC) |
| Central Bureau of Statistics (Indonesia), Ministry of Health (Indonesia), United Nations Children's Fund (UNICEF). Indonesia National Socioeconomic Survey 1995 |
| Central Bureau of Statistics (Indonesia), Ministry of Health (Indonesia), World Bank. Indonesia National Socioeconomic Survey 2001 |
| Statistics Indonesia. Indonesia National Socioeconomic Survey 2003 |
| Statistics Indonesia. Indonesia National Socioeconomic Survey 2004 |
| Statistics Indonesia. Indonesia National Socioeconomic Survey 2007 |
| Central Bureau of Statistics (Indonesia), Macro International, Inc, Ministry of Health (Indonesia), National Family Planning Coordinating Board (Indonesia). Indonesia Demographic and Health Survey 1991. Fairfax, United States of America: ICF International |
| Central Bureau of Statistics (Indonesia), Macro International, Inc, Ministry of Health (Indonesia), National Family Planning Coordinating Board (Indonesia). Indonesia Demographic and Health Survey 1994. Fairfax, United States of America: ICF International |
| Central Bureau of Statistics (Indonesia), Macro International, Inc, Ministry of Health (Indonesia), National Family Planning Coordinating Board (Indonesia). Indonesia Demographic and Health Survey 1997. Fairfax, United States of America: ICF International |
| Macro International, Inc, Ministry of Health (Indonesia), National Family Planning Coordinating Board (Indonesia), Statistics Indonesia. Indonesia Demographic and Health Survey 2002-2003. Fairfax, United States of America: ICF International |
| Macro International, Inc, Ministry of Health (Indonesia), National Family Planning Coordinating Board (Indonesia), Statistics Indonesia. Indonesia Demographic and Health Survey 2007. Fairfax, United States of America: ICF International |
| Macro International, Inc, Statistics Indonesia. Indonesia Special Demographic and Health Survey 2002-2003. Fairfax, United States of America: ICF International |
| Agency of Health Research and Development (Indonesia). Indonesia Basic Health Research 2007-2008 |
| Macro International, Inc, Statistics Indonesia. Indonesia Special Demographic and Health Survey 2007. Fairfax, United States of America: ICF International |
| Ministry of Health (Indonesia), Statistics Indonesia, United Nations Children's Fund (UNICEF). Indonesia Multiple Indicator Cluster Survey 2000. New York, United States of America: United Nations Children's Fund (UNICEF) |
| Ministry of Health (Indonesia), World Health Organization (WHO). Indonesia STEPS Noncommunicable Disease Risk Factors Survey 2001 |
| Statistics Indonesia. Indonesia National Socioeconomic Survey 2010 |
| Statistics Indonesia. Indonesia National Socioeconomic Survey 2009 |
| Minnesota Population Center, Central Bureau of Statistics (Indonesia). Indonesia Population Census 1980 from the Integrated Public Use Microdata Series, International: [Machine-readable database]. Minneapolis: University of Minnesota, 2012 |
| ICF International, Ministry of Health (Indonesia), National Population and Family Planning Board (Indonesia), Statistics Indonesia. Indonesia Demographic and Health Survey 2012. Fairfax, United States of America: ICF International |
| Agency of Health Research and Development (Indonesia), Centers for Disease Control and Prevention (CDC), Ministry of Health (Indonesia), Statistics Indonesia, World Health Organization (WHO). Indonesia Global Adult Tobacco Survey 2011. Atlanta, United States of America: Centers for Disease Control and Prevention (CDC), 2014 |
| Centers for Disease Control and Prevention (CDC), Ministry of Education (Indonesia), Ministry of Health (Indonesia), World Health Organization (WHO). Indonesia Global School-Based Student Health Survey 2007 |
| Centers for Disease Control and Prevention (CDC), World Health Organization (WHO). Indonesia Global Youth Tobacco Survey 2009. Atlanta, United States of America: Centers for Disease Control and Prevention (CDC), 2013 |
| National Narcotics Board (Indonesia), University of Indonesia. Indonesia National Survey on Drug Abuse and Illicit Drugs 2005 |
| National Institute of Health Research and Development (NIHRD), Ministry of Health (Indonesia). Analysis of National and Subnational (Regional, Province) Socioeconomic Determinants of Tobacco Use and Tobacco Related Diseases in Indonesia |
| Centers for Disease Control and Prevention (CDC), World Health Organization (WHO). Indonesia Global School-Based Student Health Survey 2015. Geneva, Switzerland: World Health Organization (WHO) |
| CDC Foundation, Centers for Disease Control and Prevention (CDC), National Institute of Health Research and Development (NIHRD) (Indonesia), World Health Organization (WHO). Indonesia Global Youth Tobacco Survey 2013-2014. Atlanta, United States of America: Centers for Disease Control and Prevention (CDC) |
| Central Bureau of Statistics (Indonesia). Indonesia National Socioeconomic Survey 2016 |
| Gallup. Indonesia World Poll 2005-2006 |
| Gallup. Indonesia World Poll 2007 |
| Gallup. Indonesia World Poll 2011 |
| Gallup. Indonesia World Poll 2012 |
| Central Bureau of Statistics (Indonesia). Indonesia National Socioeconomic Survey 2017. Jakarta, Indonesia: Central Bureau of Statistics (Indonesia), 2018 |
| Euromonitor International. Euromonitor Passport - Smoking Tobacco Statistics . London, United Kingdom: Euromonitor International |
| Ministry of Health (Laos), National Institute of Public Health (NIOPH), National Statistical Center (Laos), United Nations Children's Fund (UNICEF). Laos Multiple Indicator Cluster Survey 2000. New York, United States of America: United Nations Children's Fund (UNICEF) |
| United Nations Children's Fund (UNICEF), Department of Statistics (Laos), Ministry of Health (Laos). Laos Multiple Indicator Cluster Survey 2006. New York, United States: United Nations Children's Fund (UNICEF). |
| World Health Organization (WHO). Laos World Health Survey 2003 |
| Ministry of Education and Sports (Laos), Ministry of Health (Laos), Ministry of Planning and Investment (Laos). Laos Multiple Indicator Cluster Survey 2011-2012. New York, United States of America: United Nations Children's Fund (UNICEF), 2013 |
| Centers for Disease Control and Prevention (CDC), World Health Organization (WHO). Laos Global Youth Tobacco Survey 2011. Atlanta, United States of America: Centers for Disease Control and Prevention (CDC), 2013 |
| Centers for Disease Control and Prevention (CDC), World Health Organization (WHO). Laos Global School-Based Student Health Survey 2015 |
| Gallup. Laos World Poll 2005-2006 |
| Gallup. Laos World Poll 2011 |
| Centers for Disease Control and Prevention (CDC), World Health Organization (WHO). Laos Global Youth Tobacco Survey 2003 |
| Centers for Disease Control and Prevention (CDC), Ministry of Health (Laos), Southeast Asia Tobacco Control Alliance (SEATCA), World Health Organization (WHO). Laos Global Youth Tobacco Survey 2016. Atlanta, United States of America: Centers for Disease Control and Prevention (CDC), 2016 |
| World Health Organization (WHO). Malaysia World Health Survey 2003. Geneva, Switzerland: World Health Organization (WHO), 2005 |
| Centers for Disease Control and Prevention (CDC) and World Health Organization (WHO). Malaysia Global Youth Tobacco Survey 2003. United States: Centers for Disease Control and Prevention (CDC), 2003 |
| Ministry of Health (Malaysia), World Health Organization (WHO). Malaysia STEPS Noncommunicable Disease Risk Factors Survey 2005-2006 |
| Department of Statistics (Malaysia), Minnesota Population Center. Malaysia Population and Housing Census 1980 from the Integrated Public Use Microdata Series, International: [Machine-readable database]. Minneapolis: University of Minnesota |
| Department of Statistics (Malaysia), Minnesota Population Center. Malaysia Population and Housing Census 1991 from the Integrated Public Use Microdata Series, International: [Machine-readable database]. Minneapolis: University of Minnesota |
| Department of Statistics (Malaysia), Minnesota Population Center. Malaysia Population and Housing Census 2000 from the Integrated Public Use Microdata Series, International: [Machine-readable database]. Minneapolis: University of Minnesota |
| Centers for Disease Control and Prevention (CDC), Department of Statistics (Malaysia), Institute for Public Health, Ministry of Health (Malaysia), International Islamic University Malaysia, Johns Hopkins Bloomberg School of Public Health, Ministry of Health (Malaysia), Research Triangle Institute, Inc. (RTI), University of Malaya, World Health Organization (WHO). Malaysia Global Adult Tobacco Survey 2011 |
| Centers for Disease Control and Prevention (CDC), World Health Organization (WHO). Malaysia Global Youth Tobacco Survey 2009. Atlanta, United States of America: Centers for Disease Control and Prevention (CDC) |
| Centers for Disease Control and Prevention (CDC), Ministry of Health (Malaysia), World Health Organization (WHO). Malaysia Global School-Based Student Health Survey 2012 |
| Institute for Public Health, Ministry of Health (Malaysia). Malaysia National Health and Morbidity Survey 2015 |
| Institute for Public Health, Ministry of Health (Malaysia). Malaysia National Health and Morbidity Survey 2011 |
| Gallup. Malaysia World Poll 2005-2006 |
| Gallup. Malaysia World Poll 2007 |
| Gallup. Malaysia World Poll 2011 |
| Gallup. Malaysia World Poll 2012 |
| Family Health Development Division, Ministry of Health (Malaysia), Institute for Public Health, Ministry of Health (Malaysia), Ministry of Education (Malaysia), Ministry of Health (Malaysia). Malaysia National Health and Morbidity Survey 2017 |
| Centers for Disease Control and Prevention (CDC) and World Health Organization (WHO). Maldives Global Youth Tobacco Survey 2004. Atlanta, United States: Centers for Disease Control and Prevention (CDC) |
| ICF Macro, Ministry of Health (Maldives). Maldives Demographic and Health Survey 2009. Fairfax, United States of America: ICF International |
| Centers for Disease Control and Prevention (CDC) and World Health Organization (WHO). Maldives Global Youth Tobacco Survey 2007. United States: Centers for Disease Control and Prevention (CDC), 2007 |
| Health Protection Agency (Maldives), World Health Organization (WHO). Maldives STEPS Noncommunicable Disease Risk Factors Survey 2011 |
| Centers for Disease Control and Prevention (CDC), World Health Organization (WHO). Maldives Global Youth Tobacco Survey 2011. Atlanta, United States of America: Centers for Disease Control and Prevention (CDC) |
| Centers for Disease Control and Prevention (CDC), Ministry of Education (Maldives), Ministry of Health and Family (Maldives), World Health Organization (WHO). Maldives Global School-Based Student Health Survey 2009. Geneva, Switzerland: World Health Organization (WHO) |
| Ministry of Health and Family (Maldives). Maldives Health Statistics 2009. Malé, Maldives: Ministry of Health and Family (Maldives) |
| Ministry of Health (Maldives). Maldives Health Statistics 2013. Malé, Maldives: Ministry of Health (Maldives), 2014 |
| Centers for Disease Control and Prevention (CDC), Ministry of Education (Maldives), Ministry of Health (Maldives), World Health Organization (WHO). Maldives Global School-Based Student Health Survey 2014 |
| Centers for Disease Control and Prevention (CDC) and World Health Organization (WHO). Myanmar Global Youth Tobacco Survey 2004. Atlanta, United States: Centers for Disease Control and Prevention (CDC) |
| Ministry of Health (Myanmar), United Nations Children's Fund (UNICEF). Myanmar Multiple Indicator Cluster Survey 2000. New York, United States of America: United Nations Children's Fund (UNICEF) |
| World Health Organization (WHO). Myanmar World Health Survey 2003. Geneva, Switzerland: World Health Organization (WHO), 2005 |
| Centers for Disease Control and Prevention (CDC) and World Health Organization (WHO). Myanmar Global Youth Tobacco Survey 2001. United States: Centers for Disease Control and Prevention (CDC), 2001 |
| Centers for Disease Control and Prevention (CDC) and World Health Organization (WHO). Myanmar Global Youth Tobacco Survey 2007. United States: Centers for Disease Control and Prevention (CDC), 2007 |
| Ministry of Health (Myanmar), World Health Organization Regional Office for South-East Asia (SEARO). Myanmar STEPS Noncommunicable Disease Risk Factors Survey 2009 |
| Centers for Disease Control and Prevention (CDC), Joint United Nations Program on HIV/AIDS (UNAIDS), United Nations Children's Fund (UNICEF), United Nations Educational, Scientific and Cultural Organization (UNESCO), World Health Organization (WHO). Myanmar Global School-Based Student Health Survey 2007. Geneva, Switzerland: World Health Organization (WHO) |
| Centers for Disease Control and Prevention (CDC), World Health Organization (WHO). Myanmar Global Youth Tobacco Survey 2011. Atlanta, United States of America: Centers for Disease Control and Prevention (CDC) |
| Ministry of Health (Myanmar), World Bank, World Health Organization (WHO). Tobacco Economics in Myanmar. Washington DC, United States of America: World Bank, 2003 |
| ICF International, Ministry of Health and Sports (Myanmar). Myanmar Demographic and Health Survey 2015-2016. Fairfax, United States of America: ICF International, 2017 |
| Ministry of Health (Myanmar), Myanmar Medical Association, World Health Organization (WHO). Myanmar STEPS Noncommunicable Disease Risk Factors Survey 2014. Geneva, Switzerland: World Health Organization (WHO) |
| Gallup. Myanmar World Poll 2012 |
| Centers for Disease Control and Prevention (CDC), Ministry of Health and Sports (Myanmar), World Health Organization (WHO). Myanmar Global Youth Tobacco Survey 2016. Atlanta, United States of America: Centers for Disease Control and Prevention (CDC) |
| Centers for Disease Control and Prevention (CDC) and World Health Organization (WHO). Philippines Global Youth Tobacco Survey 2004. Atlanta, United States: Centers for Disease Control and Prevention (CDC) |
| National Statistics Office (Philippines) and United Nations Children's Fund (UNICEF). Philippines Multiple Indicator Cluster Survey 1999. New York, United States: United Nations Children's Fund (UNICEF) |
| Macro International, Inc, National Statistics Office (Philippines). Philippines Demographic and Health Survey 1993. Fairfax, United States of America: ICF International |
| Department of Health (Philippines), Macro International, Inc, National Statistics Office (Philippines). Philippines Demographic and Health Survey 1998. Fairfax, United States of America: ICF International |
| Macro International, Inc, National Statistics Office (Philippines). Philippines Demographic and Health Survey 2003. Fairfax, United States of America: ICF International |
| Macro International, Inc, National Statistics Office (Philippines). Philippines Demographic and Health Survey 2008. Fairfax, United States of America: ICF International, 2010 |
| World Health Organization (WHO). Philippines World Health Survey 2003. Geneva, Switzerland: World Health Organization (WHO), 2005 |
| CDC Foundation, Centers for Disease Control and Prevention (CDC), Department of Health (Philippines), Johns Hopkins Bloomberg School of Public Health, National Statistics Office (Philippines), Research Triangle Institute, Inc. (RTI), World Health Organization (WHO). Philippines Global Adult Tobacco Survey 2009. Atlanta, United States of America: Centers for Disease Control and Prevention (CDC) |
| Centers for Disease Control and Prevention (CDC) and World Health Organization (WHO). Philippines Global Youth Tobacco Survey 2000. United States: Centers for Disease Control and Prevention (CDC), 2000 |
| Centers for Disease Control and Prevention (CDC) and World Health Organization (WHO). Philippines Global Youth Tobacco Survey 2007. United States: Centers for Disease Control and Prevention (CDC), 2007 |
| National Statistics Office (Philippines), Minnesota Population Center. Philippines Population and Housing Census 1990 from the Integrated Public Use Microdata Series, International: [Machine-readable database]. Minneapolis: University of Minnesota |
| National Statistics Office (Philippines), Minnesota Population Center. Philippines Population Census 1995 from the Integrated Public Use Microdata Series, International: [Machine-readable database]. Minneapolis: University of Minnesota |
| National Statistics Office (Philippines), Minnesota Population Center. Philippines Population and Housing Census 2000 from the Integrated Public Use Microdata Series, International: [Machine-readable database]. Minneapolis: University of Minnesota |
| Carolina Population Center, University of North Carolina at Chapel Hill, Office of Population Studies, University of San Carlos (Philippines). Philippines - Cebu Longitudinal Health and Nutrition Survey 2002-2003. Chapel Hill, United States of America: Carolina Population Center, University of North Carolina at Chapel Hill |
| Carolina Population Center, University of North Carolina at Chapel Hill, Office of Population Studies, University of San Carlos (Philippines). Philippines - Cebu Longitudinal Health and Nutrition Survey 2004-2006. Chapel Hill, United States of America: Carolina Population Center, University of North Carolina at Chapel Hill |
| Centers for Disease Control and Prevention (CDC), World Health Organization (WHO). Philippines Global School-Based Student Health Survey 2003 . Geneva, Switzerland: World Health Organization (WHO) |
| Centers for Disease Control and Prevention (CDC), World Health Organization (WHO). Philippines Global School-Based Student Health Survey 2007. Geneva, Switzerland: World Health Organization (WHO) |
| Centers for Disease Control and Prevention (CDC), World Health Organization (WHO). Philippines Global Youth Tobacco Survey 2011. Atlanta, United States of America: Centers for Disease Control and Prevention (CDC), 2013 |
| Centers for Disease Control and Prevention (CDC), World Health Organization (WHO). Philippines Global School-Based Student Health Survey 2011 |
| ISSP Research Group (2009): International Social Survey Programme: Health and Health Care - ISSP 2011. GESIS Data Archive, Cologne. ZA5800 Data file version 3.0.0, doi:10.4232/1.12252 |
| ICF International, Philippines Statistics Authority. Philippines Demographic and Health Survey 2013. Fairfax, United States of America: ICF International, 2014 |
| Food and Nutrition Research Institute, Department of Science and Technology (Philippines). Philippines National Nutrition Survey 2013-2014 |
| CDC Foundation, Centers for Disease Control and Prevention (CDC), Department of Health (Philippines), Johns Hopkins Bloomberg School of Public Health, Philippine Statistics Authority, Research Triangle Institute, Inc. (RTI), World Health Organization (WHO). Philippines Global Adult Tobacco Survey 2015. Atlanta, United States of America: Centers for Disease Control and Prevention (CDC) |
| Centers for Disease Control and Prevention (CDC), Epidemiology Bureau, Department of Health (Philippines), World Health Organization (WHO). Philippines Global Youth Tobacco Survey 2015. Atlanta, United States of America: Centers for Disease Control and Prevention (CDC) |
| Centers for Disease Control and Prevention (CDC), World Health Organization (WHO). Philippines Global School-Based Student Health Survey 2015. Geneva, Switzerland: World Health Organization (WHO), 2020 |
| ICF International, Philippines Statistics Authority, United States Agency for International Development (USAID). Philippines Demographic and Health Survey 2017. Fairfax, United States of America: ICF International, 2018 |
| Gallup. Philippines World Poll 2005-2006 |
| Gallup. Philippines World Poll 2007 |
| Gallup. Philippines World Poll 2011 |
| Gallup. Philippines World Poll 2012 |
| Centers for Disease Control and Prevention (CDC) and World Health Organization (WHO). Sri Lanka Global Youth Tobacco Survey 2003. Atlanta, United States: Centers for Disease Control and Prevention (CDC) |
| World Health Organization (WHO). Sri Lanka World Health Survey 2003. Geneva, Switzerland: World Health Organization (WHO), 2005 |
| Centers for Disease Control and Prevention (CDC) and World Health Organization (WHO). Sri Lanka Global Youth Tobacco Survey 1999. United States: Centers for Disease Control and Prevention (CDC), 1999 |
| Centers for Disease Control and Prevention (CDC) and World Health Organization (WHO). Sri Lanka Global Youth Tobacco Survey 2007. United States: Centers for Disease Control and Prevention (CDC), 2007 |
| Ministry of Health (Sri Lanka), World Health Organization (WHO). Sri Lanka STEPS Noncommunicable Disease Risk Factors Survey 2006 |
| Centers for Disease Control and Prevention (CDC), World Health Organization (WHO). Sri Lanka Global Youth Tobacco Survey 2011. Atlanta, United States of America: Centers for Disease Control and Prevention (CDC) |
| Aarhus University, Addiction Switzerland Research Institute, Alcohol Research Group, Public Health Institute, Centre for Addiction and Mental Health (Canada), Centre for Alcohol Policy Research, Turning Point Alcohol and Drug Centre (Australia), Kettil Bruun Society for Social and Epidemiological Research on Alcohol, University of North Dakota. Sri Lanka Gender, Alcohol and Culture: An International Study (GENACIS) 2002 |
| Centers for Disease Control and Prevention (CDC), Ministry of Education (Sri Lanka), Ministry of Health, Nutrition and Indigenous Medicine (Sri Lanka), National Authority on Tobacco and Alcohol (Sri Lanka), World Health Organization Regional Office for South-East Asia (SEARO). Sri Lanka Global Youth Tobacco Survey 2015. Atlanta, United States of America: Centers for Disease Control and Prevention (CDC) |
| Department of Census and Statistics (Sri Lanka), Ministry of Health (Sri Lanka), World Health Organization (WHO), World Health Organization Regional Office for South-East Asia (SEARO). Sri Lanka STEPS Noncommunicable Disease Risk Factors Survey 2014-2015 |
| Centers for Disease Control and Prevention (CDC), World Health Organization (WHO). Sri Lanka Global School-Based Student Health Survey 2016. Geneva, Switzerland: World Health Organization (WHO), 2020 |
| Alcohol and Drug Information Centre (ADIC) (Sri Lanka), National Institute of Social Development (NISD) (Sri Lanka). Sri Lanka Spot Trend Survey on Tobacco July 2014 |
| Alcohol and Drug Information Centre (ADIC) (Sri Lanka), National Institute of Social Development (NISD) (Sri Lanka). Sri Lanka Spot Survey on Tobacco Trends December 2014 |
| Gallup. Sri Lanka World Poll 2005-2006 |
| Gallup. Sri Lanka World Poll 2011 |
| Gallup. Sri Lanka World Poll 2012 |
| Alcohol and Drug Information Centre (ADIC) (Sri Lanka), National Institute of Social Development (NISD) (Sri Lanka). Sri Lanka Tobacco Trends Spot Survey 2017 |
| Centers for Disease Control and Prevention (CDC), World Health Organization (WHO). Thailand Global Youth Tobacco Survey 2005. Atlanta, United States of America: Centers for Disease Control and Prevention (CDC) |
| National Statistical Office (Thailand), United Nations Children's Fund (UNICEF). Thailand Multiple Indicator Cluster Survey 2005-2006. New York, United States of America: United Nations Children's Fund (UNICEF) |
| Ministry of Public Health (Thailand). Thailand National Health and Examination Survey 2003-2004 |
| Baqai Institute of Diabetology and Endocrinology (BIDE) (Pakistan), Centers for Disease Control and Prevention (CDC), Faculty of Public Health at Mahidol University (Thailand), Health Systems Research Institute (Thailand), Ministry of Public Health (Thailand), National Statistical Office (Thailand), Tobacco Control Research and Knowledge Management Center (Thailand), World Health Organization (WHO). Thailand Global Adult Tobacco Survey 2009. Atlanta, United States of America: Centers for Disease Control and Prevention (CDC), 2011 |
| National Statistical Office (Thailand), Minnesota Population Center. Thailand Population and Housing Census 1980 from the Integrated Public Use Microdata Series, International: [Machine-readable database]. Minneapolis: University of Minnesota |
| National Statistical Office (Thailand), Minnesota Population Center. Thailand Population and Housing Census 1990 from the Integrated Public Use Microdata Series, International: [Machine-readable database]. Minneapolis: University of Minnesota |
| National Statistical Office (Thailand), Minnesota Population Center. Thailand Population and Housing Census 2000 from the Integrated Public Use Microdata Series, International: [Machine-readable database]. Minneapolis: University of Minnesota |
| Ministry of Public Health (Thailand). Thailand National Health Examination Survey 1991-1992 |
| Centers for Disease Control and Prevention (CDC), World Health Organization (WHO). Thailand Global Youth Tobacco Survey 2009. Atlanta, United States of America: Centers for Disease Control and Prevention (CDC) |
| National Statistical Office (Thailand). Thailand Cigarette Smoking and Drinking Behavior Survey 2001 |
| National Statistical Office (Thailand). Thailand Cigarette Smoking and Drinking Behavior Survey 2007 |
| Ministry of Health (New Zealand), Statistics New Zealand. New Zealand Health Survey 1996-1997. Wellington, New Zealand: Statistics New Zealand |
| National Statistical Office (Thailand). Thailand Cigarette Smoking and Drinking Behavior Survey 2011. Bankok, Thailand: National Statistical Office (Thailand) |
| College of Population Studies, Chulalongkorn University (Thailand), Institute for Population and Social Research, Mahidol University (Thailand), International Health Policy Program (Thailand), Ministry of Education (Thailand), Ministry of Public Health (Thailand), Ministry of Social Development and Human Security (MSDHS) (Thailand), National Health Security Office (Thailand), National Statistical Office (Thailand), Thai Health Promotion Foundation, United Nations Children's Fund (UNICEF). Thailand Multiple Indicator Cluster Survey 2012. New York, United States of America: United Nations Children's Fund (UNICEF), 2016 |
| National Statistical Office (Thailand). Thailand Survey of Cigarette Smoking Behavior 1993 |
| Ministry of Public Health (Thailand). Thailand Noncommunicable Disease and Injury Behavior Risk Surveillance Survey 2005 |
| Ministry of Public Health (Thailand). Thailand Noncommunicable Disease and Injury Behavior Risk Surveillance Survey 2007 |
| Institute for Population and Social Research, Mahidol University (Thailand), International Tobacco Control Policy Evaluation Project, Thai Health Promotion Foundation, University of Waterloo (Canada). Thailand International Tobacco Control Survey 2005 |
| Institute for Population and Social Research, Mahidol University (Thailand), International Tobacco Control Policy Evaluation Project, Thai Health Promotion Foundation, University of Waterloo (Canada). Thailand International Tobacco Control Survey 2006 |
| Action on Smoking and Health Foundation (Thailand), Centers for Disease Control and Prevention (CDC), Faculty of Public Health at Mahidol University (Thailand), Health Systems Research Institute (Thailand), Ministry of Public Health (Thailand), National Statistical Office (Thailand), Tobacco Control Research and Knowledge Management Center (Thailand), World Health Organization (WHO). Thailand Global Adult Tobacco Survey 2011 |
| Ministry of Public Health (Thailand). Thailand Noncommunicable Disease and Injury Behavior Risk Surveillance Survey 2015 |
| Centers for Disease Control and Prevention (CDC), Department of Disease Control, Ministry of Public Health (Thailand), World Health Organization (WHO). Thailand Global Youth Tobacco Survey 2015. Atlanta, United States of America: Centers for Disease Control and Prevention (CDC) |
| Gallup. Thailand World Poll 2005-2006 |
| Gallup. Thailand World Poll 2011 |
| Gallup. Thailand World Poll 2012 |
| Centers for Disease Control and Prevention (CDC) and World Health Organization (WHO). Timor-Leste Global Youth Tobacco Survey 2006. Atlanta, United States: Centers for Disease Control and Prevention (CDC) |
| National Statistics Directorate (Timor-Leste), World Bank. Timor-Leste Living Standards and Measurement Survey 2001. Washington DC, United States of America: World Bank |
| ACIL Australia Pty Ltd., Australian National University, Ministry of Health (Timor-Leste), National Statistics Directorate (Timor-Leste), University of Newcastle (Australia). Timor-Leste Demographic and Health Survey 2003. Newcastle, Australia: University of Newcastle (Australia) |
| ICF Macro, Ministry of Finance (Timor-Leste), National Statistics Directorate (Timor-Leste). Timor-Leste Demographic and Health Survey 2009-2010. Fairfax, United States of America: ICF International |
| National Statistics Directorate (Timor-Leste), World Bank. Timor-Leste Living Standards and Measurement Survey 2007-2008. Washington DC, United States of America: World Bank |
| Centers for Disease Control and Prevention (CDC), World Health Organization (WHO). Timor-Leste Global Youth Tobacco Survey 2009. Atlanta, United States of America: Centers for Disease Control and Prevention (CDC), 2013 |
| Ministry of Health (Timor-Leste), National University of East Timor, World Health Organization (WHO). Timor-Leste STEPS Noncommunicable Disease Risk Factors Survey 2014 |
| Centers for Disease Control and Prevention (CDC), World Health Organization (WHO). Timor-Leste Global School-Based Student Health Survey 2015. Geneva, Switzerland: World Health Organization (WHO) |
| Centers for Disease Control and Prevention (CDC), Ministry of Health (Timor-Leste), World Health Organization Regional Office for South-East Asia (SEARO). Timor-Leste Global Youth Tobacco Survey 2013. Atlanta, United States of America: Centers for Disease Control and Prevention (CDC) |
| ICF International, National Statistics Directorate (Timor-Leste). Timor-Leste Demographic and Health Survey 2016. Fairfax, United States of America: ICF International, 2018 |
| Centers for Disease Control and Prevention (CDC) and World Health Organization (WHO). Viet Nam Global Youth Tobacco Survey 2007. Atlanta, United States: Centers for Disease Control and Prevention (CDC) |
| World Bank (WB), General Statistics Office (Viet Nam). Viet Nam Living Standards Measurement Survey 1992-1993. Washington D.C., United States: World Bank (WB) |
| General Statistics Office (Viet Nam), United Nations Development Programme (UNDP), World Bank (WB). Viet Nam Living Standards Measurement Survey 2002. General Statistical Office, World Bank |
| General Statistics Office (Vietnam), United Nations Children's Fund (UNICEF). Vietnam Multiple Indicator Cluster Survey 2000. New York, United States of America: United Nations Children's Fund (UNICEF) |
| General Statistics Office (Vietnam), United Nations Children's Fund (UNICEF). Vietnam Multiple Indicator Cluster Survey 2006. New York, United States of America: United Nations Children's Fund (UNICEF) |
| Macro International, Inc, National Committee for Population and Family Planning (Vietnam). Vietnam Demographic and Health Survey 1997. Calverton, United States of America: Macro International, Inc |
| General Statistics Office (Vietnam), Macro International, Inc. Vietnam Demographic and Health Survey 2002. Fairfax, United States of America: ICF International |
| World Health Organization (WHO). Vietnam World Health Survey 2002-2003. Geneva, Switzerland: World Health Organization (WHO), 2005 |
| Bloomberg Philanthropies, CDC Foundation, Centers for Disease Control and Prevention (CDC), General Statistics Office (Vietnam), Hanoi Medical University, Ministry of Health (Vietnam), World Health Organization (WHO). Vietnam Global Adult Tobacco Survey 2010 |
| General Statistics Office (Vietnam), United Nations Development Programme (UNDP), World Bank. Vietnam Living Standards Measurement Survey 2006 |
| World Health Organization (WHO). Vietnam - Hồ Chí Minh STEPS Noncommunicable Disease Risk Factors Survey 2005 |
| General Statistics Office (Viet Nam), Minnesota Population Center. Viet Nam Population Census 1989 from the Integrated Public Use Microdata Series, International: [Machine-readable database]. Minneapolis: University of Minnesota |
| General Statistics Office (Viet Nam), Minnesota Population Center. Viet Nam Population and Housing Census 1999 from the Integrated Public Use Microdata Series, International: [Machine-readable database]. Minneapolis: University of Minnesota |
| General Statistics Office (Viet Nam), Minnesota Population Center. Viet Nam Population and Housing Census 2009 from the Integrated Public Use Microdata Series, International: [Machine-readable database]. Minneapolis: University of Minnesota |
| General Statistics Office (Vietnam), World Bank. Vietnam Living Standards Measurement Survey 1997-1998. Washington DC, United States of America: World Bank |
| General Statistics Office (Vietnam), United Nations Children's Fund (UNICEF). Vietnam Multiple Indicator Cluster Survey 2010-2011. New York, United States of America: United Nations Children's Fund (UNICEF) |
| Centers for Disease Control and Prevention (CDC), World Health Organization (WHO). Vietnam Global School-Based Student Health Survey 2012-2013. Geneva, Switzerland: World Health Organization (WHO) |
| General Statistics Office (Vietnam), United Nations Children's Fund (UNICEF). Vietnam Multiple Indicator Cluster Survey 2013-2014. New York, United States of America: United Nations Children's Fund (UNICEF), 2015 |
| Suzuki M, Thiem VD, Yanai H, Matsubayashi T, Yoshida LM, Tho LH, Minh TT, Anh DD, Kilgore PE, Ariyoshi K. Association of environmental tobacco smoking exposure with an increased risk of hospital admissions for pneumonia in children under 5 years of age in Vietnam. Thorax. 2009; 484-9 |
| Centers for Disease Control and Prevention (CDC), Vietnam Steering Committee on Smoking and Health (VINACOSH), World Health Organization (WHO). Vietnam Global Youth Tobacco Survey 2014. Atlanta, United States of America: Centers for Disease Control and Prevention (CDC) |
| CDC Foundation, Centers for Disease Control and Prevention (CDC), General Statistics Office (Vietnam), Hanoi Medical University, Ministry of Health (Vietnam), RTI International, Vietnam Steering Committee on Smoking and Health (VINACOSH), World Health Organization (WHO). Vietnam Global Adult Tobacco Survey 2015 |
| Gallup. Vietnam World Poll 2005-2006 |
| Gallup. Vietnam World Poll 2011 |
| Gallup. Vietnam World Poll 2012 |
| Centers for Disease Control and Prevention (CDC) and World Health Organization (WHO). Mauritius Global Youth Tobacco Survey 2003. Atlanta, United States: Centers for Disease Control and Prevention (CDC) |
| World Health Organization (WHO). Mauritius World Health Survey 2003. Geneva, Switzerland: World Health Organization (WHO), 2005 |
| Ministry of Health and Quality of Life (Mauritius), World Health Organization (WHO). Mauritius STEPS Noncommunicable Disease Risk Factors Survey 2004 |
| Centers for Disease Control and Prevention (CDC), World Health Organization (WHO). Mauritius Global Youth Tobacco Survey 2008. Atlanta, United States of America: Centers for Disease Control and Prevention (CDC) |
| Centers for Disease Control and Prevention (CDC), Ministry of Health and Quality of Life (Mauritius), World Health Organization (WHO). Mauritius Global School-Based Student Health Survey 2007 |
| Centers for Disease Control and Prevention (CDC), World Health Organization (WHO). Mauritius Global School-Based Student Health Survey 2011. Geneva, Switzerland: World Health Organization (WHO), 2014 |
| Baker IDI Heart and Diabetes Institute, Imperial College London, Ministry of Health and Quality of Life (Mauritius), National Public Health Institute (Finland), Umeå University. Mauritius Noncommunicable Disease Survey 2009 |
| Centers for Disease Control and Prevention (CDC), Ministry of Health and Quality of Life (Mauritius), World Health Organization (WHO). Mauritius Global Youth Tobacco Survey 2016. Atlanta, United States of America: Centers for Disease Control and Prevention (CDC) |
| Centers for Disease Control and Prevention (CDC) and World Health Organization (WHO). Seychelles Global Youth Tobacco Survey 2002. Atlanta, United States: Centers for Disease Control and Prevention (CDC) |
| Centers for Disease Control and Prevention (CDC) and World Health Organization (WHO). Seychelles Global Youth Tobacco Survey 2007. United States: Centers for Disease Control and Prevention (CDC), 2007 |
| World Health Organization (WHO), Ministry of Health (Seychelles), Institute of Social and Preventive Medicine, University of Lausanne (Switzerland), University Hospital Center (Switzerland). Seychelles STEPS Noncommunicable Disease Risk Factors Survey 2004 |
| Centers for Disease Control and Prevention (CDC), Joint United Nations Program on HIV/AIDS (UNAIDS), Ministry of Health (Seychelles), United Nations Children's Fund (UNICEF), United Nations Educational, Scientific and Cultural Organization (UNESCO), World Health Organization (WHO). Seychelles Global School-Based Student Health Survey 2007 . Geneva, Switzerland: World Health Organization (WHO) |
| Ministry of Health (Seychelles), World Health Organization (WHO). Seychelles STEPS Noncommunicable Disease Risk Factors Survey 2013-2014 - WHO |
| Centers for Disease Control and Prevention (CDC), Ministry of Health (Seychelles), World Health Organization (WHO). Seychelles Global Youth Tobacco Survey 2015. Atlanta, United States of America: Centers for Disease Control and Prevention (CDC) |
| Centers for Disease Control and Prevention (CDC), World Health Organization (WHO). Indonesia - Jakarta Global Youth Tobacco Survey 2000. Atlanta, United States of America: Centers for Disease Control and Prevention (CDC) |
| Centers for Disease Control and Prevention (CDC), World Health Organization (WHO). Indonesia - Jakarta Global Youth Tobacco Survey 2004 . Atlanta, United States of America: Centers for Disease Control and Prevention (CDC) |
| Ministry of Health (Indonesia), World Health Organization (WHO). Indonesia - Jawa Barat STEPS Noncommunicable Disease Risk Factors Survey 2003 |
| Ministry of Health (Indonesia), World Health Organization (WHO). Indonesia - Jawa Barat STEPS Noncommunicable Disease Risk Factors Survey 2006 |
| Ministry of Home Affairs (Indonesia), National Development Planning Agency (BAPPENAS) (Indonesia), Statistics Indonesia, United Nations Children's Fund (UNICEF). Indonesia - West Papua Multiple Indicator Cluster Survey 2011. New York, United States of America: United Nations Children's Fund (UNICEF), 2013 |
| Ministry of Home Affairs (Indonesia), National Development Planning Agency (BAPPENAS) (Indonesia), Statistics Indonesia, United Nations Children's Fund (UNICEF). Indonesia - Papua Multiple Indicator Cluster Survey 2011. New York, United States of America: United Nations Children's Fund (UNICEF), 2013 |

| **GBD 2019 data input sources for second-hand smoke, Southeast Asia** |
| --- |
| Ministry of Health (Laos), National Institute of Public Health (NIOPH), National Statistical Center (Laos), United Nations Children's Fund (UNICEF). Laos Multiple Indicator Cluster Survey 2000. New York, United States of America: United Nations Children's Fund (UNICEF) |
| United Nations Children's Fund (UNICEF), Department of Statistics (Laos), Ministry of Health (Laos). Laos Multiple Indicator Cluster Survey 2006. New York, United States: United Nations Children's Fund (UNICEF). |
| Ministry of Health (Myanmar), United Nations Children's Fund (UNICEF). Myanmar Multiple Indicator Cluster Survey 2000. New York, United States of America: United Nations Children's Fund (UNICEF) |
| National Statistics Office (Philippines) and United Nations Children's Fund (UNICEF). Philippines Multiple Indicator Cluster Survey 1999. New York, United States: United Nations Children's Fund (UNICEF) |
| National Statistical Office (Thailand), United Nations Children's Fund (UNICEF). Thailand Multiple Indicator Cluster Survey 2005-2006. New York, United States of America: United Nations Children's Fund (UNICEF) |
| National Statistics Directorate (Timor-Leste), World Bank. Timor-Leste Living Standards and Measurement Survey 2001. Washington DC, United States of America: World Bank |
| World Bank (WB), General Statistics Office (Viet Nam). Viet Nam Living Standards Measurement Survey 1992-1993. Washington D.C., United States: World Bank (WB) |
| General Statistics Office (Viet Nam), United Nations Development Programme (UNDP), World Bank (WB). Viet Nam Living Standards Measurement Survey 2002. General Statistical Office, World Bank |
| General Statistics Office (Vietnam), United Nations Children's Fund (UNICEF). Vietnam Multiple Indicator Cluster Survey 2000. New York, United States of America: United Nations Children's Fund (UNICEF) |
| General Statistics Office (Vietnam), United Nations Children's Fund (UNICEF). Vietnam Multiple Indicator Cluster Survey 2006. New York, United States of America: United Nations Children's Fund (UNICEF) |
| Macro International, Inc, Ministry of Health (Cambodia), National Institute of Statistics (Cambodia). Cambodia Demographic and Health Survey 2000. Fairfax, United States of America: ICF International |
| Macro International, Inc, National Institute of Public Health (Cambodia), National Institute of Statistics (Cambodia). Cambodia Demographic and Health Survey 2005-2006. Fairfax, United States of America: ICF International |
| Central Bureau of Statistics (Indonesia), Macro International, Inc, Ministry of Health (Indonesia), National Family Planning Coordinating Board (Indonesia). Indonesia Demographic and Health Survey 1991. Fairfax, United States of America: ICF International |
| Central Bureau of Statistics (Indonesia), Macro International, Inc, Ministry of Health (Indonesia), National Family Planning Coordinating Board (Indonesia). Indonesia Demographic and Health Survey 1994. Fairfax, United States of America: ICF International |
| Central Bureau of Statistics (Indonesia), Macro International, Inc, Ministry of Health (Indonesia), National Family Planning Coordinating Board (Indonesia). Indonesia Demographic and Health Survey 1997. Fairfax, United States of America: ICF International |
| Macro International, Inc, Ministry of Health (Indonesia), National Family Planning Coordinating Board (Indonesia), Statistics Indonesia. Indonesia Demographic and Health Survey 2002-2003. Fairfax, United States of America: ICF International |
| Macro International, Inc, Ministry of Health (Indonesia), National Family Planning Coordinating Board (Indonesia), Statistics Indonesia. Indonesia Demographic and Health Survey 2007. Fairfax, United States of America: ICF International |
| Macro International, Inc, National Statistics Office (Philippines). Philippines Demographic and Health Survey 1993. Fairfax, United States of America: ICF International |
| Department of Health (Philippines), Macro International, Inc, National Statistics Office (Philippines). Philippines Demographic and Health Survey 1998. Fairfax, United States of America: ICF International |
| Macro International, Inc, National Statistics Office (Philippines). Philippines Demographic and Health Survey 2003. Fairfax, United States of America: ICF International |
| Macro International, Inc, National Committee for Population and Family Planning (Vietnam). Vietnam Demographic and Health Survey 1997. Calverton, United States of America: Macro International, Inc |
| General Statistics Office (Vietnam), Macro International, Inc. Vietnam Demographic and Health Survey 2002. Fairfax, United States of America: ICF International |
| ICF Macro, Ministry of Finance (Timor-Leste), National Statistics Directorate (Timor-Leste). Timor-Leste Demographic and Health Survey 2009-2010. Fairfax, United States of America: ICF International |
| ICF Macro, Ministry of Health (Maldives). Maldives Demographic and Health Survey 2009. Fairfax, United States of America: ICF International |
| Macro International, Inc, National Statistics Office (Philippines). Philippines Demographic and Health Survey 2008. Fairfax, United States of America: ICF International, 2010 |
| World Health Organization (WHO). Laos World Health Survey 2003 |
| World Health Organization (WHO). Mauritius World Health Survey 2003. Geneva, Switzerland: World Health Organization (WHO), 2005 |
| World Health Organization (WHO). Philippines World Health Survey 2003. Geneva, Switzerland: World Health Organization (WHO), 2005 |
| World Health Organization (WHO). Sri Lanka World Health Survey 2003. Geneva, Switzerland: World Health Organization (WHO), 2005 |
| CDC Foundation, Centers for Disease Control and Prevention (CDC), Department of Health (Philippines), Johns Hopkins Bloomberg School of Public Health, National Statistics Office (Philippines), Research Triangle Institute, Inc. (RTI), World Health Organization (WHO). Philippines Global Adult Tobacco Survey 2009. Atlanta, United States of America: Centers for Disease Control and Prevention (CDC) |
| Baqai Institute of Diabetology and Endocrinology (BIDE) (Pakistan), Centers for Disease Control and Prevention (CDC), Faculty of Public Health at Mahidol University (Thailand), Health Systems Research Institute (Thailand), Ministry of Public Health (Thailand), National Statistical Office (Thailand), Tobacco Control Research and Knowledge Management Center (Thailand), World Health Organization (WHO). Thailand Global Adult Tobacco Survey 2009. Atlanta, United States of America: Centers for Disease Control and Prevention (CDC), 2011 |
| Bloomberg Philanthropies, CDC Foundation, Centers for Disease Control and Prevention (CDC), General Statistics Office (Vietnam), Hanoi Medical University, Ministry of Health (Vietnam), World Health Organization (WHO). Vietnam Global Adult Tobacco Survey 2010 |
| Ministry of Health (Indonesia), Statistics Indonesia, United Nations Children's Fund (UNICEF). Indonesia Multiple Indicator Cluster Survey 2000. New York, United States of America: United Nations Children's Fund (UNICEF) |
| ICF Macro, Ministry of Health (Cambodia), National Institute of Statistics (Cambodia). Cambodia Demographic and Health Survey 2010-2011. Fairfax, United States of America: ICF International |
| Department of Statistics (Malaysia), Minnesota Population Center. Malaysia Population and Housing Census 1980 from the Integrated Public Use Microdata Series, International: [Machine-readable database]. Minneapolis: University of Minnesota |
| Department of Statistics (Malaysia), Minnesota Population Center. Malaysia Population and Housing Census 1991 from the Integrated Public Use Microdata Series, International: [Machine-readable database]. Minneapolis: University of Minnesota |
| Department of Statistics (Malaysia), Minnesota Population Center. Malaysia Population and Housing Census 2000 from the Integrated Public Use Microdata Series, International: [Machine-readable database]. Minneapolis: University of Minnesota |
| National Statistics Office (Philippines), Minnesota Population Center. Philippines Population and Housing Census 1990 from the Integrated Public Use Microdata Series, International: [Machine-readable database]. Minneapolis: University of Minnesota |
| National Statistics Office (Philippines), Minnesota Population Center. Philippines Population Census 1995 from the Integrated Public Use Microdata Series, International: [Machine-readable database]. Minneapolis: University of Minnesota |
| National Statistics Office (Philippines), Minnesota Population Center. Philippines Population and Housing Census 2000 from the Integrated Public Use Microdata Series, International: [Machine-readable database]. Minneapolis: University of Minnesota |
| National Statistical Office (Thailand), Minnesota Population Center. Thailand Population and Housing Census 1980 from the Integrated Public Use Microdata Series, International: [Machine-readable database]. Minneapolis: University of Minnesota |
| National Statistical Office (Thailand), Minnesota Population Center. Thailand Population and Housing Census 1990 from the Integrated Public Use Microdata Series, International: [Machine-readable database]. Minneapolis: University of Minnesota |
| National Statistical Office (Thailand), Minnesota Population Center. Thailand Population and Housing Census 2000 from the Integrated Public Use Microdata Series, International: [Machine-readable database]. Minneapolis: University of Minnesota |
| General Statistics Office (Viet Nam), Minnesota Population Center. Viet Nam Population Census 1989 from the Integrated Public Use Microdata Series, International: [Machine-readable database]. Minneapolis: University of Minnesota |
| General Statistics Office (Viet Nam), Minnesota Population Center. Viet Nam Population and Housing Census 1999 from the Integrated Public Use Microdata Series, International: [Machine-readable database]. Minneapolis: University of Minnesota |
| General Statistics Office (Viet Nam), Minnesota Population Center. Viet Nam Population and Housing Census 2009 from the Integrated Public Use Microdata Series, International: [Machine-readable database]. Minneapolis: University of Minnesota |
| National Statistics Directorate (Timor-Leste), World Bank. Timor-Leste Living Standards and Measurement Survey 2007-2008. Washington DC, United States of America: World Bank |
| General Statistics Office (Vietnam), World Bank. Vietnam Living Standards Measurement Survey 1997-1998. Washington DC, United States of America: World Bank |
| Minnesota Population Center, Central Bureau of Statistics (Indonesia). Indonesia Population Census 1980 from the Integrated Public Use Microdata Series, International: [Machine-readable database]. Minneapolis: University of Minnesota, 2012 |
| General Statistics Office (Vietnam), United Nations Children's Fund (UNICEF). Vietnam Multiple Indicator Cluster Survey 2010-2011. New York, United States of America: United Nations Children's Fund (UNICEF) |
| ICF International, Ministry of Health (Indonesia), National Population and Family Planning Board (Indonesia), Statistics Indonesia. Indonesia Demographic and Health Survey 2012. Fairfax, United States of America: ICF International |
| Ministry of Education and Sports (Laos), Ministry of Health (Laos), Ministry of Planning and Investment (Laos). Laos Multiple Indicator Cluster Survey 2011-2012. New York, United States of America: United Nations Children's Fund (UNICEF), 2013 |
| Ministry of Home Affairs (Indonesia), National Development Planning Agency (BAPPENAS) (Indonesia), Statistics Indonesia, United Nations Children's Fund (UNICEF). Indonesia - West Papua Multiple Indicator Cluster Survey 2011. New York, United States of America: United Nations Children's Fund (UNICEF), 2013 |
| Ministry of Home Affairs (Indonesia), National Development Planning Agency (BAPPENAS) (Indonesia), Statistics Indonesia, United Nations Children's Fund (UNICEF). Indonesia - Papua Multiple Indicator Cluster Survey 2011. New York, United States of America: United Nations Children's Fund (UNICEF), 2013 |
| Agency of Health Research and Development (Indonesia), Centers for Disease Control and Prevention (CDC), Ministry of Health (Indonesia), Statistics Indonesia, World Health Organization (WHO). Indonesia Global Adult Tobacco Survey 2011. Atlanta, United States of America: Centers for Disease Control and Prevention (CDC), 2014 |
| Centers for Disease Control and Prevention (CDC), Department of Statistics (Malaysia), Institute for Public Health, Ministry of Health (Malaysia), International Islamic University Malaysia, Johns Hopkins Bloomberg School of Public Health, Ministry of Health (Malaysia), Research Triangle Institute, Inc. (RTI), University of Malaya, World Health Organization (WHO). Malaysia Global Adult Tobacco Survey 2011 |
| Ministry of Health (Cambodia), University of Health Sciences (Cambodia), World Health Organization (WHO). Cambodia STEPS Noncommunicable Disease Risk Factors Survey 2010 |
| ICF International, Philippines Statistics Authority. Philippines Demographic and Health Survey 2013. Fairfax, United States of America: ICF International, 2014 |
| College of Population Studies, Chulalongkorn University (Thailand), Institute for Population and Social Research, Mahidol University (Thailand), International Health Policy Program (Thailand), Ministry of Education (Thailand), Ministry of Public Health (Thailand), Ministry of Social Development and Human Security (MSDHS) (Thailand), National Health Security Office (Thailand), National Statistical Office (Thailand), Thai Health Promotion Foundation, United Nations Children's Fund (UNICEF). Thailand Multiple Indicator Cluster Survey 2012. New York, United States of America: United Nations Children's Fund (UNICEF), 2016 |
| General Statistics Office (Vietnam), United Nations Children's Fund (UNICEF). Vietnam Multiple Indicator Cluster Survey 2013-2014. New York, United States of America: United Nations Children's Fund (UNICEF), 2015 |
| ICF International, Ministry of Health (Cambodia), National Institute of Statistics (Cambodia). Cambodia Demographic and Health Survey 2014. Fairfax, United States of America: ICF International, 2017 |
| Suzuki M, Thiem VD, Yanai H, Matsubayashi T, Yoshida LM, Tho LH, Minh TT, Anh DD, Kilgore PE, Ariyoshi K. Association of environmental tobacco smoking exposure with an increased risk of hospital admissions for pneumonia in children under 5 years of age in Vietnam. Thorax. 2009; 484-9 |
